# Pet-directed speech draws adult dogs’ attention more efficiently than Adult-directed speech

**DOI:** 10.1038/s41598-017-04671-z

**Published:** 2017-07-10

**Authors:** Sarah Jeannin, Caroline Gilbert, Mathieu Amy, Gérard Leboucher

**Affiliations:** 1 UPL, Univ Paris Nanterre, Laboratoire Ethologie, Cognition, Développement (LECD-EA3456), F92000 Nanterre, France; 2 UMR 7179, CNRS/MNHN, Ecole Nationale Vétérinaire d’Alfort (ENVA), 94700 Maisons-Alfort, France

## Abstract

Humans speak to dogs using a special speech register called Pet-Directed Speech (PDS) which is very similar to Infant-Directed Speech (IDS) used by parents when talking to young infants. These two type of speech share prosodic features that are distinct from the typical Adult-Directed Speech (ADS): a high pitched voice and an increased pitch variation. So far, only one study has investigated the effect of PDS on dogs’ attention. We video recorded 44 adult pet dogs and 19 puppies when listening to the same phrase enounced either in ADS or in PDS or in IDS. The phrases were previously recorded and were broadcasted via a loudspeaker placed in front of the dog. The total gaze duration of the dogs toward the loudspeaker, was used as a proxy of attention. Results show that adult dogs are significantly more attentive to PDS than to ADS and that their attention significantly increases along with the rise of the fundamental frequency of human’ speech. It is likely that the exaggerated prosody of PDS is used by owners as an ostensive cue for dogs that facilitates the effectiveness of their communication, and should represent an evolutionarily determined adaptation that benefits the regulation and maintenance of their relationships.

## Introduction

Humans speak to dogs using a special speech register called pet-directed speech (PDS)^1–4^, which is very similar to infant-directed speech (IDS) used by parents when talking to young infants. These two types of speech share prosodic and syntactic features that are distinct from the typical adult-directed speech (ADS): a high pitched voice, an increased pitch variation, short utterances, a reduced syntactic and semantic complexity, and word repetitions^1–5^. PDS and IDS are also commonly described as ‘happy voices’, in comparison to ADS presenting a relatively inhibited emotional content^6^. Both speeches have been shown to vary according to the interaction context^7–9^, for instance PDS’ prosodic features are enhanced in a positive reunion situation^9^.

Several studies suggest that IDS is used by humans in order to modulate infants’ attention and state of arousal and to communicate their positive affect and intentions in a non-verbal way^10–14^. IDS may also facilitate the emergence of language in infants by emphasizing the linguistic structure^15, 16^, for instance by using hyperaticulation of vowels^2, 3, 17^, or words repetition^18^. Authors highlighted these functions in studying babies’ preference for IDS toward ADS^19, 20^: infants have a longer fixation on, or turn more often the head toward visual targets that produced IDS^19^. Infants also better remember and look longer at adults who have addressed them with IDS^20^, and this preference is present when IDS is produced by the infants’ own mother as well as by an unfamiliar mother^12, 20^. In addition, the exaggerated acoustic features of IDS elicit increased neural activity in infants, related to attentional processing^21^. Infants also present increased social and affective responsiveness while listening to IDS compared to ADS^19^. PDS and IDS may be similar because both infants and dogs are non-verbal listeners and because the affective bond between owners and dogs mirrors the human parents-infant bond. Indeed, both owners and dogs experience an important secretion of oxytocin after a brief period of cuddling^22^ and a study highlighted common brain activation when mothers viewed images of both their child and dog^23^.

In the context of human-dog communication, there is evidence that dogs present an increased neuronal activity in the auditory cortex when listening to vocalizations with positive emotional valence compared to negative or neutral emotional valence^24^. Moreover, after a greeting involving eye contact and a high pitched voice, dogs are more likely to follow the humans gaze, similarly to young children do^25, 26^. Similarly, dogs are more motivated to answer a command to find hidden food in high-pitched informative than in low-pitched imperative trials^27^, suggesting that they are sensitive to the nonverbal quality of human vocal signals.

However, while IDS has been shown to enhance attention of infants who prefer this type of speech, to our knowledge only one study has investigated dogs’ responses for PDS^28^. In their study, Ben-Aderet and coworkers exposed dogs to broadcast female voices obtained by asking women to speak in front of dogs’ pictures. They found that puppies showed a greater reaction to PDS than to ADS and were very sensitive to high frequencies^28^.

Hence, the aim of our study is to explore if PDS and IDS increase dogs’ attention to a more important extent than ADS using recording from real interactions and a large sample of dogs. We hypothesize that both puppies and adult dogs will be more attentive in response to the exacerbated prosodic features of PDS and IDS than to those of ADS, but that they will be comparably attentive to PDS and IDS prosodic and syntactic features that are distinct from ADS.

## Results

### Acoustic analyses (^*^)

There was no interaction between the recording order and the type of speech on acoustic parameters (see Table S1 in supplementary results).

There was no effect of the recording order on acoustic parameters (see Table S2 in supplementary results).

There were significant effects of the type of speech on several acoustic parameters: (a) PDS and IDS had a higher *Mean F0* than ADS (GLMM: *χ* ^*2*^_2_ = 15.50, *P < *0.001; Tukey post-hoc tests respectively *z = *4.46, *P < *0.001 and *z = *3.45, *P = *0.002). (b) *DiffES*. PDS and IDS had a wider *DiffES* than ADS (GLMM: *χ* ^*2*^_2_ *= *11.84, *P = *0.003, Tukey post-hoc tests respectively *z* = 3.00, *P = *0.007 and z = 3.60, *P < *0.001). (c) PDS and IDS had a wider *Range F0* than ADS (GLMM: *χ* ^*2*^_2_ *= *13.58, *P = *0.001, Tukey post-hoc tests respectively z = 3.24, *P = *0.003 and z = 3.99, *P < *0.001). (d) PDS and IDS had a greater *F0CV* than ADS (GLMM: *χ* ^*2*^_2_ *= *12.90, *P = *0.002, Tukey post-hoc tests respectively z = 2.73, *P = *0.018 and z = 4.01, *P < *0.001). (e) IDS had a greater *IntCV* than ADS and PDS (GLMM: *χ* ^*2*^_2_ *= *9.31, *P = *0.009, Tukey post-hoc tests respectively z = 2.72, *P = *0.018 and z = 2.96, *P = *0.008). (f) There was no significant effect of the type of speech on the speech *duration* (GLMM: *χ* ^*2*^_2_ = 3.94, *P = *0.139). Further statistical analyses were performed on other acoustic features to complete the comparison (see supplementary results).

(^*^): For each test, n = 9.

### Dogs’ behavioural response to playback

**Figure 1:**
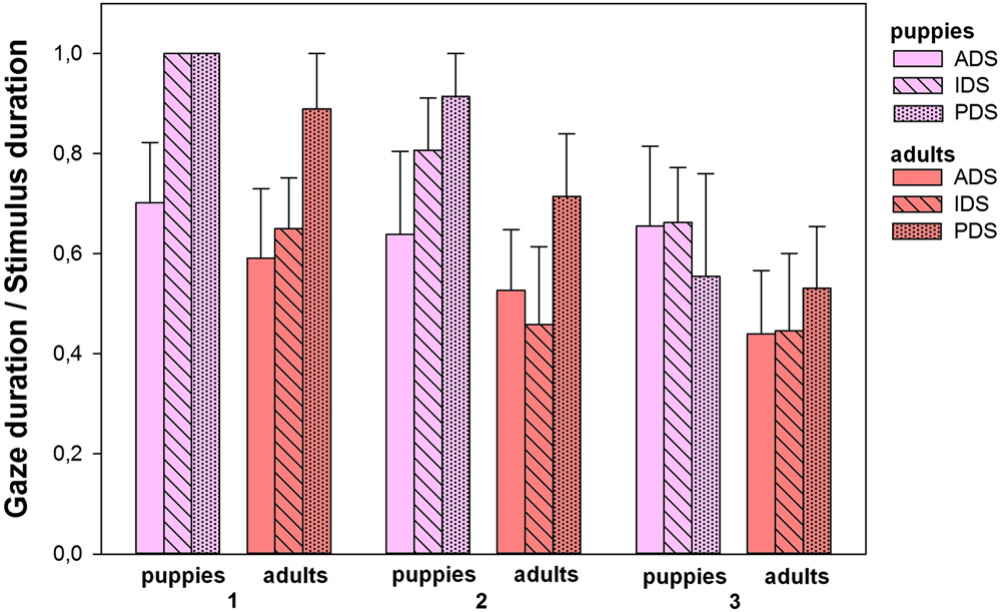
Effect of the interaction between playback order (1, 2 or 3), dogs’ age (puppies versus adult dogs) and type of speech (adult-directed, infant-directed, pet-directed) on dogs’ gaze duration toward the loudspeaker. Gaze duration is expressed as the relation between the gaze duration and the stimulus duration.

Results of the playback experiment showed that the variables ‘type of speech’, ‘playback order’ and ‘dog age’ (puppies’ gaze duration is longer than adults’ gaze duration), as well as their interaction significantly affect dogs’ behavioural response to human vocal stimuli. The other factors: the presence of children at home, the dog familiarity with people of both gender and dog sex did not significantly affect dogs’ response (Table 1). Results of the post-hoc analyses are illustrated in Fig. 1 and presented in Table 2. Only relevant comparisons were considered; we only kept situations that differed by one factor. For instance, we compared the effect of ADS in adult dogs when the speech is broadcast in first position *vs*. the effect of PDS in adult dogs when the speech is broadcast in first position; see below *Effect of the type of speech in adult dogs*.

**Table 1:** Effect of the presence of children at home, dogs’ familiarity with people of both gender, dogs’ sex, type of speech (adult-directed, infant-directed, pet-directed), playback order and dogs’ age on dogs’ behavioural response to playback.

term	estimate	s.e.	d.f.	χ²	*p*-value
children at home	−0.03	1.2	1	3.23	0.072
familiarity to gender	−1.19	1.4	1	1.19	0.276
dog sex	−2.26	1.4	1	0.13	0.722
types of speech	−0.34	1.3	8	107.26	<*0.001*
playback order	−1.93	1.3	6	122.81	<*0.001*
dog age	−2.61	1.6	6	25.97	<*0.001*
speech × order	−1.42	1.2	10	170.34	<*0.001*
speech × age	−0.37	1.3	10	117.19	<*0.001*
age × order	−2.30	1.3	9	131.56	<*0.001*
speech × order × age	−1.65	1.2	11	175.92	<*0.001*

Significant *p*-values are given in italics.

**Table 2:** Statistical analysis of data presented in Fig. 1.

	1pA	1pI	1pP	1aA	1aI	1aP	2pA	2pI	2pP	2aA	2aI	2aP	3pA	3pI	3pP	3aA	3aI	3aP	
**1pA**		**—**	**—**	**—**	**—**	**—**	**—**	**—**	**—**	**—**	**—**	**—**	**—**	**—**	**—**	**—**	**—**	**—**	**1pA**
**1pI**			**—**	**—**	**—**	**—**	**—**	**—**	**—**	**—**	**—**	**—**	**—**	**—**	**—**	**—**	**—**	**—**	**1PI**
**1pP**				**—**	**—**	**—**	**—**	**—**	**—**	**—**	**—**	**—**	**—**	**—**	**—**	**—**	**—**	**—**	**1pP**
**1aA**					**—**	**	**—**	**—**	*	**—**	**—**	**	**—**	**—**	**—**	**—**	**—**	**—**	**1aA**
**1aI**						**—**	**—**	**—**	**—**	*	**	**—**	**—**	**—**	**—**	**—**	*	**	**1aI**
**1aP**							**—**	**—**	**—**	**	**	**—**	**—**	**—**	**—**	**	**	**	**1aP**
**2pA**								**—**	**—**	**—**	**—**	**—**	**—**	**—**	**—**	**—**	**—**	**—**	**2pA**
**2pI**									**—**	**—**	**—**	**—**	**—**	**—**	**—**	**—**	**—**	**—**	**2pI**
**2pP**										*	**	**—**	*	**—**	**—**	**—**	*	**	**2pP**
**2aA**											**—**	**	**—**	**—**	**—**	**—**	**—**	**—**	**2aA**
**2aA**											**—**	**	**—**	**—**	**—**	**—**	**—**	**—**	**2aA**
**2aI**												**	**—**	**—**	**—**	**—**	**—**	**—**	**2aI**
**2aP**													**—**	**—**	**—**	**	**	**	**2aP**
**3pA**														**—**	**—**	**—**	**—**	**—**	**3pA**
**3pI**															**—**	**—**	**—**	**—**	**3pI**
**3pP**																**—**	**—**	**—**	**3pP**
**3aA**																	**—**	**—**	**3aA**
**3aI**																		**—**	**3aI**

Results from post-hoc analyses (Tukey test). Statistic interactions between playback order (1, 2 or 3), dogs’ age (p = puppies and a = adult dogs) and type of speech (A = Adult-directed, I = Infant-directed and P = Pet-directed) on dogs’ behavioural response to the playback. —NS, *p ≤ 0.05, **p ≤ 0.01.

### Effect of the acoustic parameters on dogs’ attention

The interactions between *Range F0* and dog age, *DiffES* and dog age, *Harm* and dog age did not significantly affect dogs’ response, with respectively: *χ* ^*2*^_1_ = 1.73, *P* = 0.189, *χ* ^*2*^_1_ = 1.12, *P* = 0.290 and *χ* ^*2*^_1_ = 0.19, *P* = 0.659. The interactions between (a) *Mean F0* and dog age, (b) *F0CV* and dog age, (c) *IntCV* and dog age significantly affected dogs’ behavioural response to human speeches, with respectively: *χ* ^*2*^_1_ = 4.13, *P* = 0.042, *χ* ^*2*^_1_ = 5.29, *P* = 0.021 and *χ* ^*2*^_1_ = 7.90, *P* = 0.005.

Subsequent Pearson correlation tests show that adult dogs’ attention significantly increased along with the rise of the fundamental frequency of women’ speech (Table 3). All other correlations were non-significant (Table 3).

**Table 3:** Correlations between acoustic features (Mean F0, F0CV and IntCV) and dogs’ attention for each age and for each type of speech (adult-directed, infant-directed and pet-directed speech). Pearson’s r are given with *P*-values in brackets. Significant *P*-value is in bold.

Dog age	Type of speech	Mean F0	F0CV	IntCV
Adult dogs	ADS	0.01 (0.97)	0.07 (0.65)	0.06 (0.71)
	IDS	0.07 (0.68)	0.17 (0.30)	−0.12 (0.45)
	PDS	0.37 (**0.02**)	0.27 (0.10)	−0.03 (0.86)
Puppies	ADS	0.29 (0.17)	0.13 (0.53)	0.03 (0.90)
	IDS	−0.16 (0.47)	−0.19 (0.37)	0.02 (0.91)
	PDS	−0.13 (0.55)	−0.27 (0.20)	−0.36 (0.08)

### Adult dogs

#### Effect of the type of speech in adult dogs

When considering the first broadcast stimulus we found a significant difference between PDS and ADS (Tukey test, *z* = 4.10, *P* < 0.01). Dogs’ gaze duration was longer for PDS than for ADS. In contrast, there were no significant difference between PDS and IDS on the one hand (*z* = 2.48, *P* = 0.43) and between IDS and ADS on the other hand (*z* = 2.77, *P* = 0.24). When considering the second broadcast stimulus we found significant differences between PDS and ADS on the one hand (*z* = 4.39, *P* < 0.01) and between PDS and IDS on the other hand (*z* = 5.24, *P* < 0.01). Dogs’ gaze duration was longer for PDS than for both IDS and ADS. No difference was found between ADS and IDS (*z* = −1,591, *P* = 0.97). When considering the third stimulus broadcasted, no significant difference was found in each case (*P* > 0.05) (Fig. 1 and Table 2).

#### Effect of playback order in adult dogs

When considering PDS, significant differences were found between the third and the first broadcast stimulus on the one hand (*z* = −4.67, *P* < 0.01) and between the third and the second broadcast stimulus on the other hand (*z* = −4.80, *P* < 0.01). Dogs’ gaze duration was shorter when the stimulus was broadcast in third position than both in the first and second positions. No difference was found between the first and the second broadcast stimuli (*z* = −1.496, *P* = 0.981). When considering IDS, significant differences were found between the first and second broadcast stimuli on the one hand (*z* = −4.378, *P* < 0.01) and between the first and third broadcast stimulus on the other hand (*z* = −3,384, *P* = 0.046). Dogs’ gaze duration was longer when the stimulus was broadcast in first position than both in the second and third positions. No difference was found between the second and third broadcast stimuli (*z* = 1.129, *P* = 0.999). When considering ADS, no significant difference was found in each case (*P* > 0.05) (Fig. 1 and Table 2).

### Puppies

#### Effect of the type of speech and effect of playback order in puppies

No significant difference was found (*P* > 0.05).

### Comparison between adult dogs and puppies

For each type of speech and for each playback position, we compared the gaze duration of adult dogs and puppies (for instance: ADS in the first playback position, then ADS in the second playback position etc.). No significant difference was found (*P* > 0.05).

## Discussion

As expected, adult dogs discriminated between ADS and PDS and displayed longer gaze duration when listening to PDS compared to ADS. This result disappeared when the vocal stimulus was broadcast in third position, suggesting a possible habituation phenomenon to the repetition of PDS stimuli. Regarding vocal stimuli played in first position, dogs’ responses to IDS were intermediate between responses to ADS and PDS, but the difference between IDS and PDS, as well as the difference between IDS and ADS never reached significance. If we consider that the total gaze duration is a measure of attention, as suggested by previous studies^18–20, 29, 30^, we can conclude that adult dogs are more attentive to the exaggerated PDS’s acoustic features than to ADS.

However, no significant results were found when looking at puppies’ responses to human vocal stimuli. Overall, puppies tend to show a greater reaction to all vocal stimuli compared to adult dogs as revealed by the significant effect of the ‘dog age’ variable. It is likely that puppies have a high level of attention towards human speech sounds. This particularity might slower the habituation pattern.

Furthermore, when we looked specifically to the acoustic features that interacted with dogs’ attention, we found that adult dogs’ attention increased along with the rise of the fundamental frequency of women’ speech. As previously mentioned, puppies remained alert whatever the intensity of the acoustic parameters.

In that sense, our results do not confirm the findings of Ben-Aderet *et al*.^28^ carried on twenty adult dogs and ten puppies which showed that only puppies were more attentive to PDS. This can be explained by differences in experimental protocol. For instance Ben-Aderet *et al*.^28^ recorded PDS by asking women to speak in front of pictures of dogs, instead of real interactions. Moreover they used a composite index whereas we used gaze duration as a measure of dogs’ attention. This may help to explain differences between their results and the present ones. It must be kept in mind that, as recently shown^9^, the context of the women-dog interaction significantly modulates the prosodic characteristics of PDS.

The fact that dogs discriminate between ADS and PDS is consistent with previous findings showing that dogs presented the longest gaze duration in response to a meaningful speech, i.e. a familiar command with positive intonation, while the shortest duration was in response to a meaningful speech in an unfamiliar accent with neutralized intonation^31^. These results can be explained by the fact that positive emotional valence vocalizations produce a more activated neuronal activity in dogs compared to negative or neutral emotional valence^24^ and that only praises with positive intonation activated the reward system regions^32^. So, it is likely that this specific neural activity leads to an increased attention in dogs. Indeed, similar results were found in human infants: IDS elicits increased neural activity related to attentional processing^12, 21^.

Furthermore, the preferences of human infants and dogs for exacerbated prosodic features may have an evolutionary explanation. Indeed, mammalian species used particular acoustical signals to signify motivations, intentions and emotional states that share similar acoustic features^33, 34^: high tonal sounds are associated with affiliative or submissive motivation probably because they mimic the sounds produced by infants (leading to an appeasing effect on the receiver); these sounds are generally produced in fearful or appeasing contexts^35^. For instance, dogs and wolves emit high pitched vocalizations in greeting contexts, as a solicitation for food or care^36^. In contrast, because low-frequency sounds increase the perceived size of the caller, they are generally produced in hostile contexts, during hostile interactions and associated with aggressive motivation^35^. Moreover, a study showed that the acoustic structure of particular monkey vocalizations called ‘girneys’ may be adaptively designed to attract young infants and engage their attention, similar to how the acoustic structure of human IDS, allows adults to socially engage with infants^37^. Hence, according to the authors this high pitched and musical form of speech may be biological in origin.

In contrast to our hypothesis, no significant different could be detected when comparing dogs’ responses to IDS *vs*. ADS. In addition, our results highlighted a difference between dogs’ responses to IDS and PDS when the vocal stimuli were played in second position. Moreover, when considering dogs’ response to IDS, we observed a decline in attention since the second broadcast stimulus, suggesting that dogs habituate rapidly to the repetition of IDS sequences. This difference between dogs’ responses to IDS and PDS was rather unexpected, as our acoustic analyses did not reveal any significant difference on prosodic parameters between these two types of speech. IDS only differs from PDS regarding the coefficient of variations of the intensity contour (IntCV), which suggest that speech directed to infants presents greater intensity modulation than speech directed to pets. Moreover, when analyzing the specific effect of each acoustic parameter on dogs’ attention, we found that puppies’ attention tended to decline (*P* = 0.08) with the enhancement of voice’ intensity modulation (see Table 3).

Studies of Chen and coworkers^38^ pointed out that sound intensity quantitatively contributes to emotional significance of human prosodies. They suggested that sound intensity should not simply be taken as a control parameter in neurocognitive studies of vocal emotion and that its role needs to be specified. In our study, differences regarding intensity modulation may account for differences between emotional coloring of IDS and PDS utterances. Indeed, IDS and PDS, although very similar with respect to acoustic features, are not equally perceived by human adult listeners with regards to emotional content^3^. We cannot rule out that dogs are able to perceive such differences.

On the whole, our study shows that PDS’ acoustic features elicit adult dogs’ attention significantly more than ADS. This preference for PDS may be promoted by learning: adult dogs probably learnt to associate PDS with positive greeting contexts, as PDS is exacerbated during friendly interactions^9^, and it is well established that dogs have a well-developed ability to associate prosodic cues of human speech with specific contexts^26, 27^. It is also likely that owners may use acoustic features such as pitch modulations as a tool to highlight focal words in order to enhance the dog’s comprehension and to some extent with the aim of teaching the dog some basics utterances. Such speaking strategy is used during interactions with elderly people^39^, or with linguistic foreigners^40^. From a practical perspective, our study provides support for the use of PDS by dogs’ instructors as a key tool to facilitate learning.

During communication with infants and dogs, human use ostensive signals which facilitate the communication of their intentions^41, 42^ and ‘happy talks’, like IDS and PDS, should represent an evolutionarily determined adaptation that benefits the regulation and maintenance of their relationships. The analogy between human-dog and parent-infant communication should be considered in the context of emotional relationship. The human-dog link mirrors the parent-infant bond^43^ and has been shown to share a common biological basis^22, 44, 45^.

## Methods

### Subjects

Out of the 71 pet dogs that took part into the experiment, a small proportion of subjects (n = 8) failed to react to the audio stimuli (see below for detailed information about stimuli) and were excluded from our analyses. So, participants were 63 pet dogs of various breeds, involved in the study on the basis of their owners’ volunteer participation. Forty-four adult dogs (average age of 3.74 years, range: 1 year to 14.25 years) and 19 puppies (less than one year, average age of 3.53 months, range: 2 months to 6 months) were tested (see supplementary material, Table S1 for details).

### Experimental stimuli

Nine women (age M = 31.37, SD = 10.53, see supplementary material, Table S2 for details) naive to the purpose of this study were recorded to provide audio clips of 3 different types of speech: ADS, IDS and PDS. The recordings were performed in a silent room of the laboratory Ethology Cognition and Development at the University of Nanterre. Women speaker were equipped with a lapel microphone (Olympus ME-15) connected to a MARANTZ PMD620 digital recorder. Samples were collected in ‘wav’ format with sampling frequency of 44100 Hz.

In a recent study, Ben-Aderet *et al*.^28^ obtained dog-directed speech by asking women to speak in front of dogs’ pictures. In order to provide more ecological validity to our data, our recordings were performed during interaction with real dogs, infants and adult. Thus, in a pseudo-randomized design, women were asked to address a single sentence: “On va se promener?” (“Shall we go for a stroll?”) (1) to a dog (a 2 years old Labrador Retriever, a 18 months old Chihuahua or a 18 months Maltese), (2) to an infant (either a 4 months old girl or a 3 months old girl), and (3) to the researcher (always the same) performing the recordings. The sentence was agreed in advance; we chose this sentence because it can be addressed similarly to an adult, to an infant or to a dog. There was one trial per condition (ADS, IDS, PDS). Women speaker were instructed to attract the interlocutor’s attention by saying his/her name before speaking.

**Figure 2:**
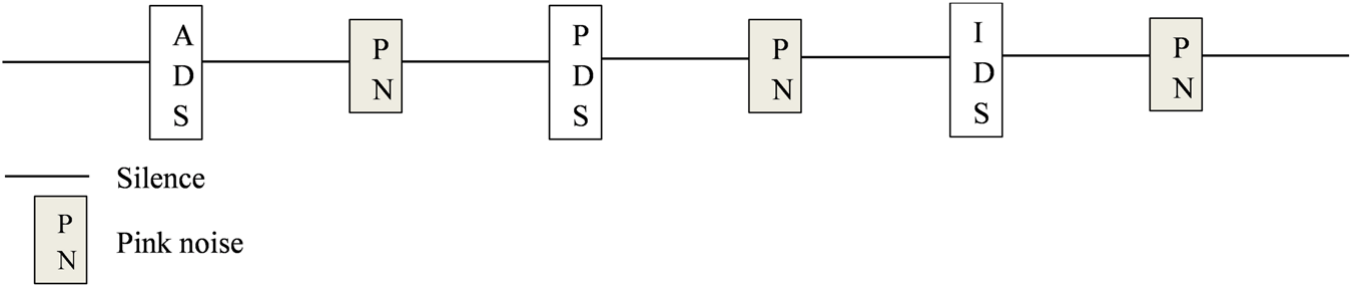
Example of an audio clip. Open squares correspond to human speeches: Adult-Directed Speech (ADS), Infant-Directed Speech (IDS) or Pet-Directed Speech (PDS) (duration: 0.69 ± 0.18 s). Grey squares correspond to pink noise (0.6 s) and lines correspond to silences (2 s).

Acoustic analyses were performed using PRAAT to ensure that IDS and PDS were distinct from ADS (see Results section). Twenty seven audio clips were created using Audacity® software corresponding to the 3 type of speech (ADS, IDS, PDS) for each of the 9 women. Each audio clip was composed by the 3 sentences, each lasting between 0.52 to 1.52 seconds depending to the speed of delivery (*X* ± SD = 0.69 ± 0.18 seconds), spoken by the same woman and separated by 2 seconds’ silence, a 0.6 second pink noise and another 2 seconds’ silence, as presented in Fig. 2. The diffusion of a pink noise between two sentences intended to attract dog’s attention and dampen the process of habituation. The order of the speeches within a clip was randomized. Each audio clip lasted between 16.72 and 17.77 seconds (*X* ± SD = 17.29 ± 0.06 seconds). Men voices were not recorded in order to match the gender of the voices diffused and the gender of the experimenter placed in front of the dog, as there is evidence that dogs can match male faces to voices^46, 47^.

### Procedure

The experiment was carried out at the Ecole Nationale Vétérinaire d’Alfort, France (ENVA). The protocols were approved by the Ethics Committee for Clinical Research (Comité d’Ethique en Recherche Clinique, ComERC) of ENVA, n° 2015-03-11. All methods were performed in accordance with the relevant guidelines and regulations. Informed consent was obtained from all participants. Participants were debriefed about the aims of the study at the end of the experiment. Seventy-one owner and dog dyads were recruited in the waiting room of the preventive medicine consultation of the Centre Hospitalier Universitaire Vétérinaire d’Alfort (CHUVA) and through veterinary students’ social networks. Participants whose dogs presented significant health problem, aggressiveness toward people, sight or hearing problems were not tested. The aim of the research was presented to the participants as follows: ‘we would like to explore what dogs perceive from human language’.

### Apparatus

The study was performed in a 24 m² room. Videos were recorded using a Canon (Legria HF R306) recorder mounted on a tripod positioned at the back-center of the room in front of a loudspeaker (Anchor MiniVox Lite) connected to a computer disposed on a table of 1 m high. The chair where the owner was sitting was aligned with the video recorder and the loudspeaker. A sonometer (Ro-Line SPL meter, R0-1350) was used to measure the sound intensity: all sounds were 90 decibels.

### Experimental protocol

**Figure 3:**
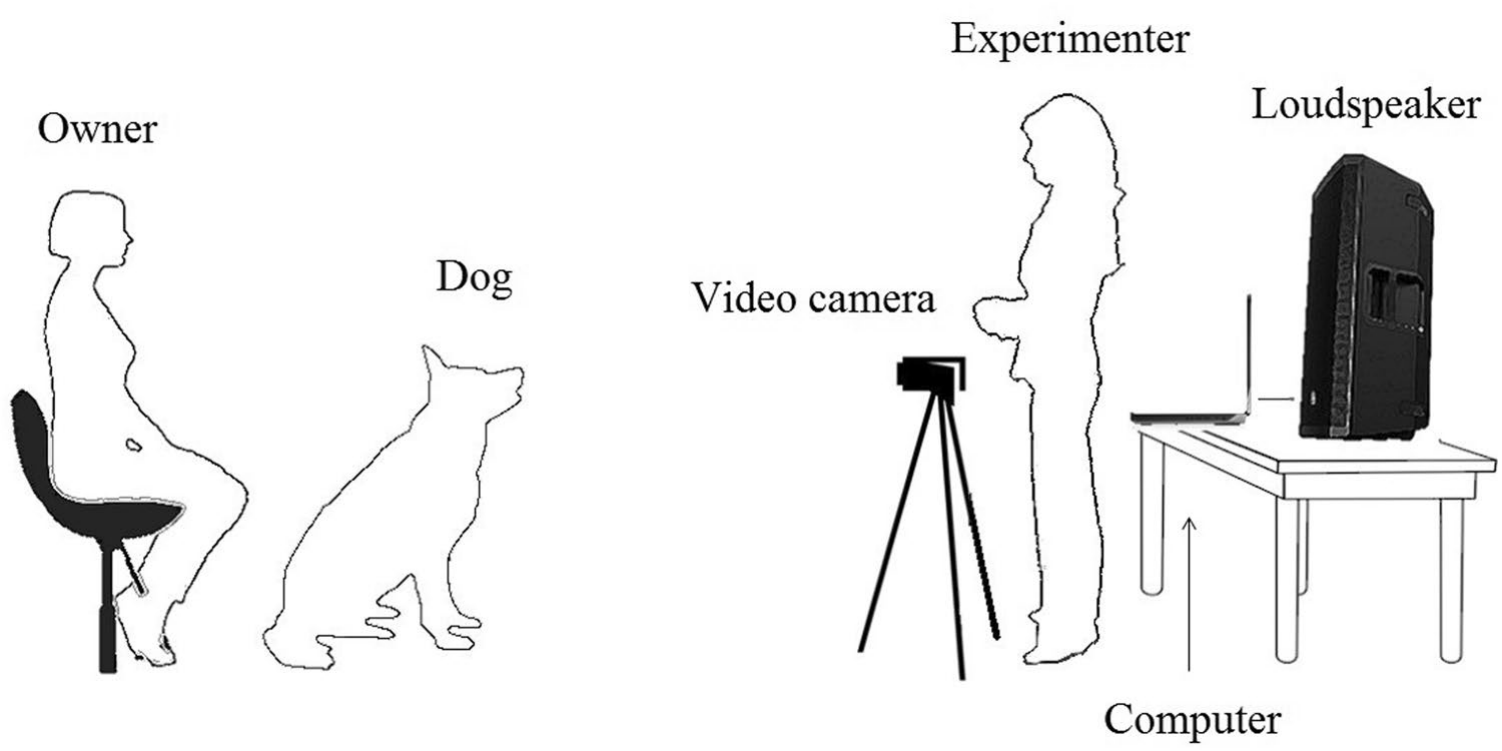
Schematic drawing of the setup.

Initially, the owner was asked about his/her dog’s name, age and breed, composition of the family etc., while the dog was let free to explore the room. Then he/she was invited to sit on a chair placed in front of the video recorder and to install his/her dog between his/her legs or to put him on his/her knees. Because prior studies found that dogs often ignore vocal commands given by humans (or recordings of humans) if no human is physically present^48, 49^, a female experimenter (S.J.) was constantly present, standing in front of the loudspeaker in order to increase the likelihood that the dog would pay attention to the vocal recordings played by the loudspeaker (Fig. 3). The experimenter adjusted the video image for each dog so that the dog was in the central part of the image. Blinded to the playback order, the experiment launched the audio sequence, using the computer behind her, when the dog was calm and well positioned, using the computer behind her. Few seconds of silence were programmed before the playback starts to allow time for the experimenter to position and remain immobile. The experimenter looked straight in front of her to avoid eye contact with the dog. Owners were asked not to speak or stroke the dog during the playback. Each dog listened to one randomly selected audio sequence.

### Data analyses

#### Acoustic analyses

In order to verify that the recordings used for the playbacks had characteristics properties of ADS, IDS or PDS, acoustic analyses were performed using a script Praat software (5.3.50)^50^. We treated each recording as one continuous vocalization; the analysis was made on utterance level. We measured the following parameters: (a) *duration*: the total duration of the recording; (b) *Mean F0*: the average fundamental frequency F0 calculated over the duration of the signal; (c) *Diff ES*: the difference between mean F0 at the end of the recording and mean F0 at the start of the recording. *Diff ES* is considered to be an indicator of the intonation contour; (d) *Range F0*: the range of the fundamental frequency F0; (e) *F0CV*: the coefficient of variation of F0 over the duration of the signal, estimated as the standard deviation of F0 divided by mean F0; (f) *IntCV*: the coefficient of variation of the intensity contour.

#### Additional acoustic analyses

Further analyses were made on supplementary parameters (harmonicity, shimmer, jitter, the first three formant frequencies of the vocal stimuli). Moreover, because previous studies showed a significant difference between IDS, PDS and ADS based on vowel hyperarticulation^3^, we measured this parameter using Andruski *et al*.’ procedure^51^; vowel hyperarticulation was objectified by plotting first and second formant (F1 and F2) values of the determinant vowels of the sentence “on va se promener?”: a [a], o [o], and é [e], and comparing the resultant vowel triangles (see supplementary results, Figure S1 for details). The acoustic space encompassed by the 3 point vowel categories was compared by calculating the area of the vowel triangle for each type of speech. Vowel triangle area was calculated as: 1/2*[X1(Y2-Y3) + X2(Y3-Y1) + X3 (Y1-Y2)] where X and Y are the mean F1 and F2 values, and 1, 2, and 3 are the point vowels, [a], [o], and [e].

#### Video coding

Dogs’ behavioural response to human vocal stimuli was recorded and analyzed with Solomon Coder software (version beta 16.06.26). We made continuous observations from videos with a time-precise of one-tenth of a second. For each dog and for each vocal stimulus (ADS, IDS, and PDS), we measured the total duration of looks toward the loudspeaker, referred as “gaze duration” in our statistical analysis. This measure is the standard one used to assess infants’ attention^18, 19^. Moreover, it was used in previous studies to assess dogs’ attention and effect of various conditions on this parameter (i.e. aging^29^; relationship^30^; oxytocin^22^). In order to take the variability of stimulus duration (from 0.52 to 1.52 seconds) gaze duration toward the loudspeaker, was expressed as the relation between the gaze duration and the stimulus duration.

### Statistical analyses

#### Acoustic analyses

The normality of the data was tested using Shapiro-Wilk tests. To test whether the recording order and the type of speech influenced the acoustic features of women speakers, we used general linear mixed models (GLMMs) using library ‘lme4’ of the R software. For each acoustic feature, we constructed model with the type of speech, the recording order and their interaction as fixed effects and the identity of the speaker as random effects. We first tested the model with the interaction against the model without the interaction. If the interaction was no significant, we removed it from the model and then tested one-factor models (either type of speech or recording order) against the minimal model. Finally, we performed Tukey post hoc tests when appropriated, using library ‘multcomp’ of the R software; Tukey test was developed specifically to account for multiple comparison and maintains experiment-wise alpha at the specified level (0.05)^52^. Statistics were performed using R© version 3.2.4 (The R foundation for statistical computing, Vienna, Austria).

#### Analysis of dogs’ behavioural response to playback

To test for differences in type of speech on dogs’ behaviour, we used GLMM with the glmer function of the package ‘lme4’ using R© version 3.2.4. GLMMs allowed us to build a model with both fixed effects and random effects, and to specify data distribution (here a binomial distribution). To take into account the variation of the duration of the stimuli, ‘Gaze duration’ divided by ‘Stimulus duration’ was set as the dependent variable; ‘Stimulus duration’ was thus specified as a ‘weights’ argument in the model. The dog’s identity was specified as the random factor to control for repeated measures. Dog familiarity with people of both gender referred as “dog familiarity to gender” (women only, men only, both sexes), the presence of children at home (yes or no), dog age (adult *vs*. puppy), dog sex (male *vs*. female), type of speech (ADS, IDS, PDS), playback order (first, second or third position) and their interaction were specified as the fixed factors. Likelihood-ratio tests were performed to obtain P values by comparing the full models with reduced models (without the fixed effect). If appropriate, these analyses were followed by Tukey post hoc tests using the glht function of the package ‘multcomp’.

Moreover, to assess how the different acoustic features affected dogs’ attention, we built a model for each acoustic feature that significantly differentiated ADS from IDS and PDS. Hence, the acoustic feature, dog age, their interactions and playback order were specified as fixed factors. As in previous analyses, ‘Gaze duration’ divided by ‘Stimulus duration’ was set as the dependent variable (‘Stimulus duration’ was specified as a ‘weights’ argument in the model). The dog’s identity was specified as the random factor to control for repeated measures. P values were obtained with likelihood-ratio tests comparing the models with interaction and models without interaction.

Significant interactions were followed by Pearson correlations in order to assess the relationship between acoustic features and dogs’ attention for each age (adult dogs and puppies) and for each type of speech (ADS, IDS and PDS).

### Ethical approval

The study received the approval of the ethical committee of ENVA (COMERC), 477 n° 2015-03-11.

## Electronic supplementary material

Supplementary material

Data file playback experiment

Data file acoustic analyses
