# Supplementary material for: Pet-directed speech draws adult dogs’ attention more efficiently than Adult-directed speech
Source: Sci Rep. 2017 Jul 10;7:4980. doi: 10.1038/s41598-017-04671-z (PMC5504008; doi:10.1038/s41598-017-04671-z)
Supplement: Supplementary file 1 — Supplementary material [file 41598_2017_4671_MOESM1_ESM.pdf]

# Pet-directed speech draws adult dogs' attention more efficiently than Adult-directed speech

Sarah Jeannin*, Caroline Gilbert, Mathieu Amy, Gérard Leboucher

## Supplementary Material

**Supplementary Table S3**. Characteristics of the dogs tested during the playback experiments

| Name | Age (months) | Sexe | Breed | Familiarity with gender | Children at home |
|---|---|---|---|---|---|
| Nikita | 98 | Male | Shih Tzu | only women | No |
| Iwak | 16 | Female | Miniature dachshund | both sexes | No |
| Youkha | 78 | Female | Labrador Retriever | only women | No |
| Loki | 3 | Male | French bulldog | both sexes | No |
| Izzie | 12 | Female | Chihuahua | only women | Yes |
| Ines | 12 | Female | Chihuahua | only women | Yes |
| Gotchat | 37 | Female | Australian Shepherd | both sexes | No |
| Gina | 36 | Female | French bulldog | only women | |
| Iden | 4 | Male | American staff | both sexes | No |
| Jedi | 3 | Male | Labrador Retriever | only women | No |
| Hemalin | 19 | Male | Australian Shepherd | both sexes | No |
| Indy | 12 | Female | Staff-bull terrier | only women | No |
| Enjie | 55 | Female | Australian Kelpie | both sexes | Yes |
| Goss-bo | 39 | Male | Picardy Shepherd | only women | Yes |
| Soca | 32 | Female | French bulldog | only men | No |
| Vicky | 114 | Female | Cavalier King Charles | both sexes | No |
| Griotte | 31 | Female | Cavalier King Charles | both sexes | No |
| Hindi | 20 | Female | Cavalier King Charles | both sexes | Yes |
| Bibi | 24 | Male | Yorkshire Terrier | only women | No |
| Iroy | 4 | Male | Golden retriever | only men | No |
| Maya | 84 | Female | Labrador Retriever Mix | only women | |
| Pinch | 84 | Male | Pinscher | only women | |
| Lili | 39 | Female | Chihuahua | both sexes | No |
| Jocker | 3 | Male | American staff | only women | |
| Iatiss | 5 | Female | Labrador Retriever | both sexes | No |
| Ivy | 4 | Female | Japanese Spitz | only women | |
| Italie | 5 | Female | Labrador Retriever | both sexes | No |

| | | | | | |
|---|---|---|---|---|---|
| Hello Iota | 18 | Female | White Swiss Shepherd | both sexes | No |
| Inkyo | 10 | Male | Australian Cattle | both sexes | No |
| Gribouille | 171 | Male | Terrier Mix | only women | |
| Havann | 26 | Female | Yorkshire Terrier | both sexes | Yes |
| Gesshoku | 4 | Female | Labrador Retriever | both sexes | No |
| Piao | 15 | Male | Terrier Mix | both sexes | No |
| Goliath | 3 | Male | Jack Russel Mix | only women | Yes |
| Ipsos | 12 | Male | Picardy Spaniel | only men | |
| India | 6 | Female | Shih Tzu | only women | Yes |
| Mia | 90 | Female | Labrador Retriever | only women | |
| Geo | 33 | Male | Yorkshire Terrier | only women | |
| Luna | 96 | Female | Husky | only women | No |
| Yanka | 16 | Female | Husky | only women | |
| Wendy | 3 | Female | English Coker | only women | |
| Blue | 7 | Male | Cane Corso | only women | |
| Gitan | 16 | Male | Presa Canario Dog | both sexes | Yes |
| Ikane | 15 | Female | Presa Canario Dog | both sexes | Yes |
| Jeep | 3 | Female | Bernese Mountain Dog | both sexes | No |
| Felie | 36 | Female | English Cocker | both sexes | Yes |
| Laika | 120 | Female | Rottweiler Mix | both sexes | Yes |
| Ely | 48 | Male | Parson Russell Terrier | only women | No |
| Caline | 72 | Female | Griffon Mix | only women | No |
| Folie | 50 | Female | Labrador Retriever | only women | Yes |
| Jango | 3 | Male | French bulldog | only women | |
| Lucas | 2 | Male | Rottweiler | both sexes | No |
| Singha | 132 | Female | Pug | only women | No |
| Cana | 48 | Female | Terrier Mix | only women | No |
| Pirate | 2 | Female | Jack Russel | only women | |
| Viton | 24 | Male | Yorkshire Terrier | only women | Yes |
| Irko | 4 | Male | Golden Retriever | both sexes | No |
| Lucky | 7 | Male | Yorkshire Terrier | only women | No |
| Diky | 7 | Male | Yorkshire Terrier | only women | Yes |
| Kheldar | 13 | Male | Belgian Shepherd Mix | both sexes | No |
| Cooky | 50 | Male | Japanese Spitz Mix | only women | No |
| Jedi | 3 | Male | Argentine dogo | both sexes | Yes |
| Jaisy | 3 | Female | Argentine dogo | both sexes | Yes |

**Supplementary Table S4.** Characteristics of the women speaker (nicknames were changed to ensure anonymity of participants)

| Name | Age (years) | Children | Dog owner (is/was) |
|---|---|---|---|
| Aude | 24 | No | No |
| Diana | 22 | Yes | Yes |
| Doriane | 43 | Yes | Yes |
| Elisa | 22 | No | No |
| Noémie | 43 | Yes | Yes |
| Pénélope | 22 | No | Yes |
| Rosanna | 30 | Yes | Yes |
| Angela | 32 | No | No |
| Valentine | 45 | Yes | Yes |
